# Alcohol use during pregnancy: the impact of social determinants of health on alcohol consumption among pregnant women

**DOI:** 10.1186/s13293-025-00731-6

**Published:** 2025-07-01

**Authors:** MacKenzie R. Peltier, Terril L. Verplaetse, Vera Bici, Abbie A. Mokwuah, C. Leonard Jimenez Chavez, Yasmin Zakiniaeiz, Robert Kohler, Vernon Garcia-Rivas, Bubu A. Banini, Hang Zhou, Nakul R. Raval, Brian Pittman, Sherry A. McKee

**Affiliations:** 1 Yale School of Medicine, Department of Psychiatry, 40 Temple Street Suite: 5B, New Haven, CT 06519 USA; 2 VA Connecticut Healthcare System, Mental Health Service, West Haven, CT 06516 USA; 3 Clinical Neuroscience Division, National Center of PTSD, West Haven, CT 06516 USA; 4 Yale School of Medicine, Section of Digestive Diseases, New Haven, CT 06519 USA

**Keywords:** SDoH, Alcohol use, Problematic drinking, Pregnancy, Trimester

## Abstract

**Background:**

There is no safe amount or time of alcohol consumption during pregnancy; however, many women drink while pregnant placing themselves and their fetuses at risk for alcohol-related health complications. Social Determinants of Health (SDoH) impact alcohol use during pregnancy. Understanding the impact of SDoH across pregnancy will elucidate important information to reduce rates of prenatal alcohol exposure.

**Methods:**

Cross-sectional data from the National Survey of Drug Use and Health from 2009 to 2019 was used to explore the impact of SDoH on alcohol use across pregnancy. The study assesses past month alcohol use and past month binge drinking, as well as various SDoH. The sample included 8,638 pregnant women.

**Results:**

Over 9% of pregnant women reported alcohol use within the past 30 days and 5.25% reported drinking on three or more days within the past month. 3.65% reported past month binge drinking. Past month alcohol use and past month binge drinking decreased in the second and third trimesters; however, a subset of women continued alcohol use, including binge drinking. Specific SDoH emerged as increasing the likelihood of alcohol use within the past month, including not being married (ORs = 1.54 to 1.94), criminal justice involvement (arrested and booked; OR = 1.88), and past year psychiatric distress (OR = 1.86). Conversely, other determinants were associated with a lower likelihood of alcohol use, including identifying as Asian or Hispanic (ORs = 0.41 and 0.64) and unemployment (OR = 0.52) and other employment (OR = 0.66). Older age was associated with a lower likelihood of binge drinking within the past month (ORs = 0.32). Unique SDoH emerged when examining alcohol use by trimester.

**Conclusion:**

Specific SDoH (i.e., not married, criminal justice involvement, past year psychiatric distress) are related to increased alcohol use during pregnancy, while other determinants (i.e., identifying as Asian or Hispanic, not requiring full time/part-time employment) are associated with a decreased risk for past month alcohol use. Older individuals (35–49 years old) and those with a high school education had a decreased likelihood of binge drinking. Accordingly, healthcare providers should screen all pregnant women for alcohol use throughout pregnancy, especially among populations or groups identified as being vulnerable to continue alcohol use during pregnancy.

## Plain language summary

There is no safe amount of or time for alcohol use during pregnancy; however, many women will continue to drink during pregnancy, placing both themselves and their fetus at increased risk for health consequences. Social Determinants of Health (SDoH), or nonmedical factors that impact one’s physical well-being, may help identify populations that would benefit from additional prenatal support and prevention efforts as related to alcohol use. The present study used ten years of publicly available, epidemiological data to examine the impact of SDoH on alcohol use across pregnancy. While alcohol use did decrease in the second and third trimesters, unique SDoH were related to past month alcohol use and binge drinking. For instance, not being married, criminal justice involvement, and past year psychiatric distress all increased the likelihood of past month alcohol use. Conversely, identifying as Asian or Hispanic and reporting unemployment or “other” employment were associated with being less likely to drink within the past month. These factors illustrate that it is important to continue to alcohol screen pregnant women across all trimesters, particularly those who are vulnerable to alcohol use during pregnancy.

## Highlights

There is no safe alcohol consumption during pregnancy; however, a subset of women continues to drink alcohol.

Compared to the first trimester, alcohol use decreases in the second and third trimesters.

Specific Social Determinants of Health (SDoH) including being married, criminal justice involvement, and past year psychiatric distress increased the likelihood to drink within the past month alcohol use.

Conversely, identifying as Asian or Hispanic and reporting unemployment or “other” employment were associated with being less likely to drink within the past month.

Healthcare providers are encouraged to screen all women for alcohol use across pregnancy, including women who are vulnerable to alcohol use during pregnancy.

## Background

There is no known safe time period or amount of alcohol to consume during pregnancy; however, many women in the United States (US) drink while pregnant. Recent data from the Behavioral Risk Factor Surveillance Survey indicates that 13.5% of pregnant women, corresponding to nearly 1 in 7, report currently drinking alcohol within the past month and of those who report current alcohol use, over 5% endorse binge drinking (consuming four or more drinks on at least one occasion) [1]. While many women stop or reduce alcohol use once they find out they are pregnant, particularly in the second and third trimesters, there remains a subset of women who continue to drink [2, 3]. This is problematic, as alcohol exposure during pregnancy is harmful to both the neonate and pregnant woman. Alcohol exposure during pregnancy increases risk of delivery complications, miscarriage, preterm delivery, and sudden infant death syndrome [4]. Additionally, alcohol use during pregnancy is associated with fetal alcohol spectrum disorders (FASDs) and is the leading cause of birth defects and neurodevelopmental abnormalities in the US. FASDs affects between 1 and 5% of first graders [5] and encompasses a wide range of behavioral, cognitive, and physical deficits [4]. However, despite these established concerns, drinking during pregnancy appears to be on the rise in the US [1, 6] and drinking among women continues to increase [7]. Ongoing alcohol use during pregnancy underscores the need to better understand who is at risk for continued alcohol use, to inform targeted prevention and intervention programs.

Social Determinants of Health (SDoH) are nonmedical influences, such as income, education, and employment, that impact health conditions. The World Health Organization (WHO) estimates that between 30 and 55% of health outcomes can be accounted for by SDoH and identifying such variables may improve health [8]. Identification of SDoH can lead to understanding and addressing health disparities, expanding the knowledge base surrounding a health problem, and improving overall living conditions [8]. For instance, previous research has illustrated that individual-level factors such as education and income, as well as community-level factors, such as health insurance and arrest history can impact alcohol use outcomes [9, 10]. Similarly, factors such as race and ethnicity, criminal justice involvement, and housing problems are related to treatment outcomes for alcohol use [10]. 

The relationship between alcohol use and SDoH is particularly salient for women [11], as women are more vulnerable to negative health outcomes associated with SDoH [12]. This is illustrated by increased adverse pregnancy-related health outcomes, including maternal mortality, among those with high SDoH burden. For instance, Black, American Indian, and Alaska Native pregnant women are at increased risk for maternal death [13], as are those with a lower socioeconomic status, and those living in rural populations [12]. Additionally, chronic exposure to such stressful determinants is related to pregnancy complications [12]. Socioeconomic status [2, 14], age [14, 15], education [16], marital status [2], and race [17] have been associated with increased risk of alcohol use during pregnancy. Given the evidence that SDoH impact alcohol use during pregnancy, it is imperative to examine the impact of various SDoH across pregnancy in order to highlight populations that may benefit from additional prenatal support and prevention efforts as related to alcohol use.

### The present study

The present study sought to examine the impact of SDoH on alcohol use among pregnant women across trimesters using ten years of data from a representative, national dataset. It was hypothesized that alcohol use, including both past month alcohol use and past month binge drinking, would decrease as pregnancy progressed from the first trimester to the second and third trimesters. Additionally, it was predicted that unique SDoH, specifically socioeconomic status (employment and income), age, race/ethnicity, education, marital status, criminal justice involvement, health insurance, geographical location, and stress would be related to prenatal alcohol use.

## Methods

### Study data

The present study used cross-sectional, annual data from the 2009–2019 National Survey on Drug Use and Health (NSDUH). This national survey was administered by the Substance Abuse and Mental Health Services Administration (SAMSHA) to collect data describing the substance and alcohol use, demographic, and health-related factors of noninstitutionalized US civilians. The sample included 8,638 pregnant women. Additional description of the survey and its methodology is available [18]. 

### Measures

Pregnancy. Pregnancy was assessed through self-report survey by asking survey respondents if they were pregnant and if so, to identify the current trimester of their pregnancy.

SDoH variables. SDoH variables included age, education, marital status, race/ethnicity, employment status, income, survey language, metro size, and overall health. Also, the following dichotomous variables were assessed: receipt of government programs, health insurance, arrest history, mental health treatment in the past year, lifetime major depressive disorder (MDD), and past year psychiatric distress (utilizing the Kessler-6 (K6) distress scale for the worst month within the past year). Responses for race/ethnicity were recoded to create groups large enough to include in the models described below (Native American/Alaskan Native, Native Hawaiian/Other Pacific Islander, and more than one race were categorized as “Other”). Additionally in 2015, survey questions assessing marital status, education, employment, and metro size were altered and given new variable names in the subsequent annual surveys. These variables were recoded to identically match the datasets in the preceding and subsequent survey iterations. In the present study, variables from years 2009–2014 were combined with equivalent marital status, education, employment, and metro size variables from 2015 to 2019 [18]. 

Alcohol use. Recent alcohol use, past month alcohol use, past month binge drinking, and number of days of alcohol within the past month use were used to assess alcohol use across pregnancy. Prior to 2015, NSDUH defined binge drinking as consuming five or more drinks on the same occasion regardless of sex; however, in 2015 binge drinking was redefined as four or more drinks on the same occasion for women. The present study coded these binge drinking variables into one variable, despite the change in definition to illustrate binge alcohol use in pregnancy [18]. 

### Statistical analyses

SDoH and alcohol use were calculated for pregnant women overall and separately by trimester individually to examine changes across pregnancy. Logistic regressions examined the impact of SDoH factors on past month alcohol use and past month binge drinking among the sample of women identifying as pregnant. Additionally, a logistic regression examined the likelihood of past month alcohol use across trimesters. A model predicting past month binge drinking across individual trimesters was not included a due to small sample sizes. Each model included all SDoH factors allowing for simultaneous adjustment. All analyses accounted for the NSDUH survey design, by incorporating stratification, clustering (i.e., primary sampling unit), and weightings using survey procedures (e.g., PROC SURVEYLOGISTIC) in SAS, version 9.4 (Cary, NC).

## Results

### Participants

 Of the 8,638 women self-identifying as pregnant, 2,659 women identified as being in the first trimester, 3,112 in the second trimester, and 2,759 in the third trimester. The majority of the women were 26–34 years old (49.46%) and college graduates (33.48%). Over half of the participants were married (59.40%) and identified as White (56.75%). Most were employed full-time (42.24%), earned either between $20,000–49,999 (30.64%) or over $75,000 per year (31.24%), reported being in “very good” health (38.60%), and lived in a large metro area (53.96%). A majority of the sample took the survey in English (94.50%), had health insurance (92.13%), and did not receive government programs (70%). A small portion of the sample endorsed a history of criminal justice involvement (i.e., being arrested and booked; 13.41%), had past year mental health treatment (12.88%), and had a lifetime history of MDD (14.07%). For completed results and results by trimester, see Table  1.

**Table 1:** NSDUH (2009–2019; *n* = 8,638 pregnant individuals) weighted sociodemographic variables

	Pregnant Individuals (n = 8,638)	First Trimester	Second Trimester	Third Trimester
		(n = 2,659)	(n = 3,112)	(n = 2,759)
SDoH				
Age				
12–17 years old	2.36%	2.84%	2.14%	1.75%
18–25 years old	33.92%	32.24%	33.82%	35.64%
26–34 years old	49.46%	51.01%	48.29%	50.11%
34–49 years old	14.26%	13.91%	15.75%	12.50%
Education				
Less than high school	15.91%	15.33%	16.09%	15.63%
High school graduate	23.92%	24.10%	23.76%	23.63%
Some college	26.69%	26.35%	26.11%	28.08%
College graduate	33.48%	34.23%	34.05%	32.66%
Marital Status				
Married	59.40%	57.86%	60.14%	60.76%
Widowed/Divorced	5.76%	7.90%	4.83%	4.71%
Single	34.85%	34.25%	35.03%	34.53%
Race				
White	56.75%	57.50%	56.18%	57.79%
Black	15.08%	16.45%	14.38%	14.41%
Asian	6.08%	6.36%	5.59%	6.12%
Hispanic	19.15%	16.58%	20.53%	19.26%
Other	2.95%	3.11%	3.31%	2.42%
Employment Status				
Full-time	42.42%	47.39%	41.75%	38.68%
Part-time	17.14%	15.68%	17.46%	18.30%
Unemployed	7.01%	9.66%	7.01%	4.28%
Other	33.43%	27.27%	33.79%	38.74%
Income				
<$20,000	21.39%	21.55%	21.17%	21.20%
$20,000–49,999	30.64%	29.53%	30.34%	31.71%
$50,000–74,999	16.73%	16.76%	17.35%	16.38%
>$75,000	31.24%	32.16%	31.14%	30.72%
Survey Language				
English	94.50%	95.12%	94.69%	94.08%
Spanish	5.50%	4.88%	5.31%	5.92%
Government Programs				
No	70.00%	69.29%	70.10%	70.58%
Yes	30.01%	30.71%	29.90%	29.42%
Health Insurance				
No	7.88%	11.57%	6.92%	5.22%
Yes	92.13%	88.43%	93.09%	94.78%
Arrested/Booked				
No	86.59%	85.97%	86.89%	86.85%
Yes	13.41%	14.03%	13.12%	13.15%
MH Treatment in PY				
No	87.12%	85.20%	87.66%	88.22%
Yes	12.88%	14.80%	12.34%	11.78%
Lifetime MDD				
No	85.93%	84.25%	86.03%	87.13%
Yes	14.07%	15.75%	13.97%	12.87%
PY Psychiatric Distress				
No	87.40%	84.92%	87.49%	89.53%
Yes	12.60%	15.08%	12.51%	10.47%
Metrosize				
Large	53.96%	55.11%	54.15%	52.11%
Small	31.60%	30.77%	32.03%	32.40%
Nonmetro	14.44%	14.12%	13.82%	15.49%
Overall Health				
Excellent	32.63%	31.17%	31.56%	35.31%
Very Good	38.60%	38.28%	40.28%	37.29%
Good	23.52%	23.54%	22.73%	24.17%
Fair/Poor	5.25%	7.01%	5.43%	3.24%

Note. SDoH = Social Determinates of Health; PY = past year; MDD = Major Depressive Disorder.

### Alcohol use

The majority of pregnant women (52.68%) endorsed consuming alcohol more than 30 days prior, but within the past 12 months. Additionally, 9.47% reported alcohol use within the past month. A total of 3.65% reported binge drinking within the past month and 5.25% reported drinking on three or more days within the past month. See Table 2 for the distributions by trimester.

**Table 2:** NSDUH (2009–2019) weighted drinking variables

	Pregnant Individuals (*n* = 8,638)	First Trimester (*n* = 2,659)	Second Trimester (*n* = 3,112)	Third Trimester (*n* = 2,759)
Recent alcohol use				
Never	16.04%	16.39%	14.73%	16.14%
Within 30 days	9.47%	19.77%	5.55%	3.28%
> 30 days, but within past 12 months	52.68%	53.93%	55.16%	49.97%
>12 months	21.82%	9.91%	24.56%	30.61%
Past month alcohol use				
Yes	9.47%	19.77%	5.55%	3.28%
No	90.54%	80.23%	94.45%	96.72%
Past month binge* drinking				
Yes	3.65%	8.92%	1.59%	0.48%
No	96.36%	91.08%	98.41%	99.52%
Past month days of drinking				
None	90.54%	80.23%	94.45%	96.72%
1–2 days	4.22%	7.58%	2.78%	2.22%
3–5 days	2.99%	6.92%	1.32%	0.83%
6–19 days	1.86%	4.33%	1.23%	0.17%
20–30 days	0.40%	0.95%	0.21%	0.07%

Note. *NSDUH data prior to 2015 defined binge drinking as > 5 drinks per occasion regardless of sex; whereas data from 2015-19 defined binge drinking among women as > 4 drinks per occasion

### SDoH and alcohol use

The likelihood of alcohol use and binge drinking decreased as pregnancy progressed. In the second and third trimesters, there was a lower likelihood of past month alcohol use (ORs = 0.25 and 0.15, respectively) and past month binge drinking (ORs = 0.18 and 0.06, respectively). Identifying as not married was associated with an increased likelihood of alcohol use. Those identifying as widowed/divorced or single were almost two times more likely to have endorsed past month alcohol use (ORs = 1.54 and 1.94, respectively) and were over two to four times more likely to have reported past month binge drinking (ORs = 4.38 and 2.46, respectively) as compared to those identifying as married. Endorsing a history of being arrested/booked or past year psychiatric distress were also associated with increased odds of past month alcohol use (ORs = 1.88 and 1.86) and past month binge drinking (ORs = 1.94 and 2.03). Additionally, employment status was associated with past month alcohol use, in that those identifying as unemployed or “other” had lower odds of past month alcohol use (ORs = 0.52 and 0.66) than those working full-time. Age and education level were related to past month binge drinking. Specifically, those 34–49 years were less likely to binge drink in the past month compared to those 18–25 years (OR = 0.32) and high school graduates were less likely than those not completing high school to endorse past month binge drinking (OR = 0.61). See Table 3 for full results.

**Table 3:** Weighted odds of past month alcohol use and binge drinking

	Past month alcohol use			Past month binge* drinking		
SDoH	OR	p	95% CI	OR	p	95% CI
Trimester						
First	REF	< 0.001		REF	< 0.001	
Second	0.25	< 0.001	0.19–0.33	0.18	< 0.001	0.12–0.25
Third	0.15	< 0.001	0.11–0.22	0.06	< 0.001	0.03–0.11
Age†						
18–25 years old	REF	0.08		REF	0.01	
26–34 years old	0.99	0.94	0.74–1.33	0.7	0.09	0.46–1.06
34–49 years old	1.39	0.05	1.00-1.92	0.32	0.004	0.15–0.70
Education						
Less than high school	REF	0.52		REF	0.001	
High school graduate	0.95	0.79	0.66–1.37	0.61	0.05	0.37–0.99
Some college	1.13	0.57	0.74–1.73	0.73	0.2	0.44–1.19
College graduate	1.31	0.29	0.79–2.17	1.59	0.2	0.79–3.19
Marital Status						
Married	REF	0.01		REF	< 0.001	
Widowed/Divorced	1.94	0.01	1.18–3.20	4.38	< 0.001	2.17–8.84
Single	1.54	0.01	1.12–2.12	2.46	0.001	1.44–4.22
Race/Ethnicity						
White	REF	0.03		REF	0.36	
Black	1.21	0.22	0.89–1.64	1.47	0.09	0.94–2.30
Asian	0.41	0.03	0.18–0.92	0.6	0.42	0.17–2.16
Hispanic	0.64	0.03	0.43–0.96	0.96	0.88	0.54–1.69
Other	1.1	0.75	0.62–1.95	1.41	0.28	0.76–2.60
Employment Status						
Full-time	REF	0.002		REF	0.08	
Part-time	0.78	0.08	0.59–1.03	0.74	0.18	0.48–1.15
Unemployed	0.52	0.001	0.36–0.77	0.53	0.02	0.31–0.89
Other	0.66	0.003	0.50–0.86	0.68	0.13	0.42–1.12
Income						
<$20,000	REF	0.06		REF	0.86	
$20,000–49,999	1.38	0.04	1.02–1.85	1.08	0.69	0.74–1.58
$50,000–74,999	1.52	0.05	1.00-2.31	1.07	0.83	0.59–1.94
>$75,000	1.64	0.01	1.11–2.43	1.23	0.41	0.74–2.04
Survey Language						
English	REF			REF		
Spanish	0.73	0.46	0.31–1.70	0.7	0.61	0.18–2.78
Government Programs						
No	REF			REF		
Yes	1.12	0.49	0.82–1.53	1.12	0.54	0.77–1.64
Health Insurance						
No	REF			REF		
Yes	0.82	0.27	0.58–1.17	0.98	0.93	0.62–1.56
Arrested/Booked						
No	REF			REF		
Yes	1.88	< 0.001	1.38–2.55	1.94	0.001	1.32–2.86
MH Treatment in PY						
No	REF			REF		
Yes	1.07	0.71	0.76–1.50	1.11	0.61	0.74–1.69
Lifetime MDD						
No	REF			REF		
Yes	0.86	0.39	0.62–1.21	1	0.99	0.61–1.64
PY Psychiatric Distress						
No	REF			REF		
Yes	1.86	< 0.001	1.36–2.54	2.03	< 0.001	1.41–2.91
Metrosize						
Large	REF	0.09		REF	0.32	
Small	0.94	0.63	0.72–1.23	1.22	0.28	0.85–1.76
Nonmetro	0.71	0.03	0.51–0.97	1.38	0.16	0.88–2.18
Overall Health						
Excellent	REF	0.87		REF	0.19	
Very Good	0.99	0.92	0.75–1.30	1.03	0.89	0.69–1.54
Good	0.98	0.9	0.69–1.38	1.02	0.95	0.63–1.64
Fair/Poor	1.21	0.46	0.73–2.01	1.88	0.04	1.03–3.43

Note. SDoH = Social Determinates of Health; †=12–18 year old not included; PY = past year; MDD = Major Depressive Disorder; *NSDUH data prior to 2015 defined binge drinking as > 5 drinks per occasion regardless of sex; whereas data from 2015-19 defined binge drinking among women as > 4 drinks per occasion

### Past month alcohol use and trimester

First trimester. Older pregnant women (34–49 years old) were more likely to report alcohol use in the past month than those 18–25 years old (OR = 1.86). Also, women identifying as single were more likely to drink within the past month (OR = 1.75) as compared to those who identified as married. Those endorsing a history of criminal justice involvement and psychiatric distress within the past year were twice as likely to report past month alcohol use (ORs = 2.16 and 2.40). Conversely, women identifying as Asian (OR = 0.37) or Hispanic (OR = 0.56) and those identifying as either unemployed (OR = 0.50) or “other” employment (OR = 0.52) were less likely to drink than their counterparts identifying as White or having full-time employment, respectively.

Second trimester. Women in the second trimester were more likely to report past month alcohol use if they identified as being previously married as compared to those who were married (OR = 2.94). Race/ethnicity was significantly related to drinking within the past thirty days, with women identifying as Black being at increased risk of alcohol use (OR = 2.02), whereas women identifying as Asian were less likely to report drinking (OR = 0.003).

 Third trimester. Among those in the third trimester, women earning more than $20,000 (ORs ranged from 4.62 to 6.36) and those with health insurance (OR = 19.82) were at increased risk to report past month alcohol use. However, women identifying as Black (OR = 0.25) or Hispanic (OR = 0.32) were less likely to report alcohol use than their White counterparts and those living in non- or small metro areas (ORs 0.25 and 0.46) were also less likely to consume alcohol within the past month than those living in large metro areas. See Table  4 for complete results.

**Table 4:** Weighted odds of past month alcohol use by trimester

	First Trimester			Second Trimester			Third Trimester		
SDoH	OR	p	95% CI	OR	p	95% CI	OR	p	95% CI
Age†									
18–25 years old	REF	0.03		REF	0.84		REF	0.91	
26–34 years old	1.05	0.83	0.70–1.57	0.86	0.55	0.51–1.43	1.15	0.75	0.54–2.45
34–49 years old	1.86	0.01	1.14–3.04	0.90	0.79	0.42–1.95	1.25	0.70	0.40–3.85
Education									
Less than high school	REF	0.45		REF	0.30		REF	0.46	
High school graduate	1.13	0.61	0.71–1.82	0.78	0.56	0.33–1.82	0.64	0.36	0.24–1.69
Some college	1.43	0.13	0.90–2.29	0.83	0.68	0.35–1.98	0.62	0.33	0.23–1.65
College graduate	1.45	0.24	0.78–2.70	1.31	0.64	0.43–3.98	1.03	0.97	0.27–3.99
Marital Status									
Married	REF	0.05		REF	0.05		REF	0.30	
Widowed/Divorced	1.58	0.18	0.81–3.10	2.94	0.01	1.26–6.88	2.83	0.19	0.60-13.24
Single	1.75	0.01	1.12–2.73	1.14	0.66	0.64–2.02	1.70	0.23	0.71–4.06
Race/Ethnicity									
White	REF	0.03		REF	< 0.0001		REF	0.01	
Black	1.26	0.23	0.86–1.83	2.02	0.03	1.08–3.79	0.25	0.002	0.11–0.60
Asian	0.37	0.05	0.14-1.00	0.003	< 0.0001	< 0.001–0.02	0.95	0.94	0.27–3.34
Hispanic	0.56	0.03	0.33–0.95	1.09	0.79	0.55–2.16	0.32	0.02	0.12–0.83
Other	0.92	0.81	0.44–1.90	1.27	0.65	0.45–3.57	1.28	0.75	0.27–5.93
Employment Status									
Full-time	REF	< 0.01		REF	0.35		REF	0.14	
Part-time	0.86	0.40	0.60–1.23	0.88	0.69	0.47–1.66	0.32	0.03	0.12–0.88
Unemployed	0.50	0.003	0.32–0.78	0.50	0.08	0.23–1.08	0.40	0.28	0.07–2.17
Other	0.52	0.004	0.33–0.80	0.79	0.42	0.45–1.40	0.85	0.66	0.40–1.78
Income									
<$20,000	REF	0.54		REF	0.33		REF	0.01	
$20,000–49,999	1.14	0.53	0.76–1.70	1.11	0.74	0.60–2.07	6.36	< 0.001	2.23–17.85
$50,000–74,999	1.21	0.51	0.69–2.10	1.97	0.11	0.85–4.54	5.33	0.02	1.30-21.93
>$75,000	1.43	0.15	0.88–2.34	1.74	0.14	0.83–3.65	4.62	0.04	1.10-19.48
Survey Language									
English	REF			REF			REF		
Spanish	1.12	0.85	0.36–3.51	0.43	0.24	0.10–1.79	0.42	0.36	0.07–2.69
Government Programs									
No	REF			REF			REF		
Yes	0.95	0.78	0.63–1.41	1.26	0.39	0.74–2.15	2.13	0.23	0.63–7.23
Health Insurance									
No	REF			REF			REF		
Yes	0.75	0.19	0.48–1.16	0.88	0.75	0.41–1.89	19.82	< 0.001	4.18–93.90
Arrested/Booked									
No	REF			REF			REF		
Yes	2.16	< 0.001	1.51–3.11	1.73	0.06	0.98–3.06	1.13	0.78	0.47–2.73
MH Treatment in PY									
No	REF			REF			REF		
Yes	0.88	0.54	0.57–1.34	1.54	0.22	0.77–3.10	1.42	0.39	0.63–3.17
Lifetime MDD									
No	REF			REF			REF		
Yes	0.68	0.12	0.42–1.11	0.94	0.76	0.51–1.65	1.39	0.37	0.67–2.88
PY Psychiatric Distress									
No	REF			REF			REF		
Yes	2.40	< 0.001	1.67–3.43	1.48	0.16	0.86–2.57	1.65	0.21	0.75–3.61
Metrosize									
Large	REF	0.28		REF	0.86		REF	0.01	
Small	1.13	0.47	0.81–1.58	0.88	0.64	0.50–1.53	0.46	0.03	0.23–0.91
Nonmetro	0.82	0.32	0.55–1.22	0.86	0.67	0.43–1.73	0.25	0.01	0.09–0.69
Overall Health									
Excellent	REF	0.65		REF	0.47		REF	0.50	
Very Good	1.07	0.67	0.78–1.48	0.95	0.87	0.55–1.67	0.63	0.19	0.31–1.26
Good	0.84	0.43	0.53–1.31	1.37	0.41	0.64–2.93	0.96	0.93	0.36–2.54
Fair/Poor	1.10	0.73	0.63–1.92	1.72	0.24	0.69–4.31	0.62	0.66	0.07–5.26

Note. SDoH = Social Determinates of Health; †=12–18 year old not included; PY = past year; MDD = Major Depressive Disorder

## Discussion

The present study sought to examine the impact of specific SDoH on alcohol use among pregnant women, including within each trimester. Overall, alcohol use decreased in the second and third trimesters of pregnancy; however, specific SDoH emerged as increasing the likelihood of alcohol use within the past month, while other determinants were associated with a lower likelihood of alcohol use. For instance, not being married, criminal justice involvement, and past year psychiatric distress, were all related to increased alcohol use, while older age, identifying as Asian or Hispanic, high school completion, and non-full-time employment decreased the likelihood for alcohol use. Unique SDoH emerged when exploring alcohol use across trimesters. Within each trimester, specific determinants appeared to be protective against past month alcohol use, including being within the age range of 34–49 years old, identifying as Black, Asian, or Hispanic, working less than full-time, or living in a small or non-metro area. Conversely factors such as, identifying as previously married, earning over $20,000 per year, endorsing past criminal justice involvement, or experiencing psychiatric distress within the past year heightened the risk of past month alcohol use. However, these associations were not consistent across all trimesters, warranting further research.

The decreased likelihood of both past month alcohol use and past month binge drinking in the second and third trimesters is congruent with previous findings. Previous research reports that 87% of pregnant women stopped alcohol use during pregnancy and more than 6% reduce their drinking [19]. This is largely unsurprising given the extensive guidance from health professional organizations, including the American College of Obstetricians and Gynecologists (ACOG) [20], and federal health institutions, such as the Center of Disease Control [4] and the National Institute of Alcohol Abuse and Alcoholism [21], warning about the dangers of prenatal alcohol exposure. Additionally, many pregnant women report being motivated to stop drinking for the health of their fetus [22]. 

The present study also identified important SDoH that were associated with alcohol use during pregnancy. For example, older women were more likely to report past month alcohol use during the first trimester; however, older age also was protective against binge drinking in the overall sample. This is concerning as there is no safe level of alcohol consumption during pregnancy. Previous data has also highlighted the relationship between age and alcohol use during pregnancy, with older women being more likely to drink while pregnant and younger women being at higher risk for hazardous drinking patterns [23, 24]. Finally, the present study also suggest high school graduates were less likely than those not graduating high school to binge drink within the past month during pregnancy.

There is evidence that drinking during pregnancy is related to pre-pregnancy drinking patterns; women who report drinking before pregnancy have been shown to be four times as likely to drink during pregnancy and twice as likely to binge drink during this time [25]. Similarly, women who endorsed binge drinking at least three months prior to pregnancy were over eight times more likely to consume any alcohol while pregnant and almost 40 times more likely to continue binge drinking while pregnant [25]. Epidemiological data shows significant increases in drinking among women of reproductive age, including heightened binge drinking in women under 25 years old and those not graduating high school [7, 26, 27]. Interestingly, other SDoH, including those residing in small or nonmetropolitan areas were less likely to drink alcohol during the third trimester, despite increases in alcohol use among these groups [27]. Thus, additional research should examine how drinking increases in reproductive aged women may impact alcohol use during pregnancy.

When exploring SDoH and alcohol use during pregnancy, Asian and Hispanic identities were less likely to report past month alcohol use when compared to non-Hispanic, White women; however, this relationship was unclear across trimesters for women identifying as White. Identifying as Asian decreased the likelihood of past month drinking during the first and second trimesters and identifying as Hispanic was associated with a decreased likelihood of drinking during the first and third trimesters. Interestingly, women identifying as Black were twice as likely to reporting alcohol use during the second trimester but were less likely to report past month alcohol use in the third trimester. Previous epidemiological data has reported that Black, Asian, and Hispanic mothers are less likely to drink than non-Hispanic, White women during pregnancy; [25] however, it is unclear why women identifying as Black may be more likely to drink during the second trimester. This observation underscores the importance of healthcare providers continuously screening for alcohol use during a pregnancy and not just at the first visit regardless of endorsement of alcohol use, as these results illustrate that there may be unique nonmedical factors contributing to alcohol use at each trimester.

Similar to previous studies, pregnant women in NSDUH who were not married had an increased risk for past month drinking and past month binge drinking [2, 24, 28]. During pregnancy, psychosocial support from marital partners is related to overall improved well-being and familial support [29]. One meta-analysis demonstrated that a lack of support from a partner during pregnancy is related to negative affect, including depression and anxiety [30], which are well established risk factors for higher alcohol use in women [31]. Thus, adequate psychosocial support from a marriage may be protective against alcohol use during pregnancy. Healthcare providers should help pregnant women identify a supportive social network that can assist in coping with negative affect during pregnancy and the postpartum period.

Stress appears to have a strong relationship to alcohol use during pregnancy. In the present study, past year psychiatric distress was associated with a heightened likelihood of past month alcohol use and binge drinking, specifically among women in the first trimester. A substantial body of evidence suggests that women are more likely than men to drink to alleviate stress and cope with negative affect [31]. Consequently, women experiencing substantial past year psychiatric distress may be at greater risk for drinking during pregnancy, potentially to cope with negative affect. This also is likely related to the increased risk of past month alcohol use and binge drinking among those endorsing a history of criminal justice involvement (e.g., being arrested and booked). Women involved in criminal justice often report experiencing heightened levels of stress-related mental health disorders, including mood, anxiety, and trauma-related disorders [32] and general legal problems have also been associated with alcohol use disorder [33]. Additionally, many states have punitive laws regarding fetal substance exposure, including alcohol use [34], which may heighten stress and anxiety among pregnant women with prior criminal justice involvement due to fear of negative legal consequences related to alcohol use during pregnancy.

Surprisingly, lifetime MDD was not related to alcohol use during pregnancy. In recent years, significant efforts have been made to improve screening for and treatment of depression among pregnant women [35–38], as depression during pregnancy has been associated with adverse health outcomes, including increased instances of alcohol use [39]. Thus, efforts to emphasize the importance of depression screening and appropriate treatment of depressive disorders and mood-related symptoms may impact the effect of a depressive disorders on current alcohol use. Nonetheless, additional research to disentangle the differences between perceived psychiatric distress and a MDD diagnosis is warranted.

 Pregnant women who did not work full-time were less likely to drink within the past month. This is consistent with previous evidence demonstrating that women with higher incomes are more at risk for drinking during pregnancy [ 2, 25 ]. This was particularly evidence for women in the third trimester of pregnancy, who were four to six times more likely to report drinking in the past month compared to those earning less than $20,000. Additionally, women reporting having health insurance were also at heightened risk. Epidemiological data has shown significant increases in binge drinking among women aged 30–49 years old identifying within a high socioeconomic group, compared to the increases observed in lower income groups [ 40 ]. Thus, pregnant women who are working full-time, and thus likely earning a higher income, are at increased risk for alcohol use. There is a discrepancy between alcohol-related health consequences and socioeconomic status; while individuals in a higher income group drink at least as much as those in lower income groups, women in lower income groups experienced more alcohol-related health consequences [ 41 ]. Accordingly, providing appropriate psychosocial education regarding the dangers of drinking during pregnancy may be beneficial to pregnant women with higher incomes, who may not be acutely exposed to the health consequences of alcohol use in their own experiences.

Overall, the present study supports the current guidance from the ACOG and the US Preventive Services Task Force to screen all pregnant women for alcohol use [42]. These results also illustrate the importance of continuing alcohol screening throughout pregnancy to identify and assist the subset of women who continue to consume alcohol during pregnancy. Data shows that the use of standardized measures such as the T-ACE (Tolerance, Annoyance, Cut Down, Eye-Opener), Alcohol Use Disorder Identification Test-C (AUDIT-C) and TWEAK (Tolerance, Worried, Eye-Opener, Amnesia, “Kut” Down) are effective assessment tools to identify women at risk for drinking [43]. Appropriately screening across SDoH provides the opportunity for all pregnant women to receive education, as well as brief, counseling based interventions in order to receive the appropriate level of assistance to improve both their and their fetus’ health. Such efforts are critically important, as pregnant women with a history of alcohol use are at higher risk for prenatal alcohol exposure and drinking rates among women are at historically high levels.

### Limitations

While the present study utilized a large, national epidemiological dataset the cross-sectional nature of the survey design used in NSDUH precludes causal inferences between pregnancy and alcohol consumption. Despite its large size, some of the stratified groups exhibited small sample sizes which may have impacted the results. Thus, future research designed to examine the direct impact of SDoH on alcohol use across pregnancy would be beneficial. Furthermore, the present study is limited to the publicly available, self-reported data from the NSDUH combine 2002–2019 data file, thus introducing potential bias related to self-reporting alcohol use during pregnancy. Additionally, the variables included in the survey also limited the inclusion of other factors of interest related to SDoH, including lifetime, anxiety disorders, immigration status or religious beliefs, which may have been related to alcohol use during the perinatal period. Additionally, NSDUH does not include any assessment of alcohol use that corresponds with the current trimester or start of pregnancy. Thus, the present study utilized variables related to past month use, which may overlap with time periods prior to pregnancy, before the individual knew they were pregnant, or between trimesters. Furthermore, the survey design did not permit exploration of alcohol use during previous pregnancies. Also, the variable for binge drinking prior to 2015 did not differentiate between men and women (e.g., five drinks vs. four drinks per occasion in definition). Considering these limitations, the present study should be viewed as a conservative estimate of prenatal alcohol exposure; however, the present findings closely align with previously published rates [44], providing further support for existing evidence.

## Conclusion

While the majority of women stop drinking during pregnancy, there remains a subset who continue alcohol consumption, including binge alcohol use and specific SDoH emerged as consistent and strong factors that were driving results. Individuals who were not married, had criminal justice involvement, or had past year psychiatric distress were at increased risk for alcohol use during pregnancy. Conversely, identifying as Asian or Hispanic, or not requiring full time/part-time employment were associated with a decreased risk of past month alcohol use. Those who were older (35–49 years old) and those with a high school education had a decreased likelihood of binge drinking. These findings highlight a complex interplay between SDoH and alcohol consumption during pregnancy, identifying groups that are at increased and decreased risk. Accordingly, healthcare providers are encouraged to continuously screen pregnant women across all trimesters, particularly among populations of the groups identified as being vulnerable to alcohol use during pregnancy.

## Data Availability

Data is publicly available. Additional information can be found at https://www.samhsa.gov/data/data-we-collect.
